# An Improvised Deep-Learning-Based Mask R-CNN Model for Laryngeal Cancer Detection Using CT Images

**DOI:** 10.3390/s22228834

**Published:** 2022-11-15

**Authors:** Pravat Kumar Sahoo, Sushruta Mishra, Ranjit Panigrahi, Akash Kumar Bhoi, Paolo Barsocchi

**Affiliations:** 1 School of Computer Engineering, KIIT Deemed to be University, Bhubaneswar 751024, India; 2 Department of Computer Applications, Sikkim Manipal Institute of Technology, Sikkim Manipal University, Majitar, Rangpo 737136, India; 3 KIET Group of Institutions, Delhi-NCR, Ghaziabad 201206, India; 4 Directorate of Research, Sikkim Manipal University, Gangtok 737102, India; 5 Institute of Information Science and Technologies, National Research Council, 56124 Pisa, Italy

**Keywords:** clinicians, deep learning, ImageNet, laryngeal cancer, patients

## Abstract

Recently, laryngeal cancer cases have increased drastically across the globe. Accurate treatment for laryngeal cancer is intricate, especially in the later stages. This type of cancer is an intricate malignancy inside the head and neck area of patients. In recent years, diverse diagnosis approaches and tools have been developed by researchers for helping clinical experts to identify laryngeal cancer effectively. However, these existing tools and approaches have diverse issues related to performance constraints such as lower accuracy in the identification of laryngeal cancer in the initial stage, more computational complexity, and large time consumption in patient screening. In this paper, the authors present a novel and enhanced deep-learning-based Mask R-CNN model for the identification of laryngeal cancer and its related symptoms by utilizing diverse image datasets and CT images in real time. Furthermore, our suggested model is capable of capturing and detecting minor malignancies of the larynx portion in a significant and faster manner in the real-time screening of patients, and it saves time for the clinicians, allowing for more patient screening every day. The outcome of the suggested model is enhanced and pragmatic and obtained an accuracy of 98.99%, precision of 98.99%, F1 score of 97.99%, and recall of 96.79% on the ImageNet dataset. Several studies have been performed in recent years on laryngeal cancer detection by using diverse approaches from researchers. For the future, there are vigorous opportunities for further research to investigate new approaches for laryngeal cancer detection by utilizing diverse and large dataset images.

## 1. Introduction

Laryngeal cancer is becoming a serious threat in people’s lives in the modern era across the globe. Laryngeal cancer has been found to be one of the critical malignancies within the neck and head regions in people [1]. Cases of laryngeal cancer are increasing rapidly among people every year all around the world. The clinical cure prognosis and alternatives rely on the stage of cancer at the instance of identification of laryngeal cancer [2]. The initial phase of laryngeal cancer and pre-cancer is linked with higher degrees of laryngeal preservation along with a positive diagnosis in real time [3], while advanced levels of laryngeal cancer demand a multi-modal diagnosis plan, which results in vital toxicities and poor life quality. Regarding an optimized diagnosis plan, existing research demonstrates a higher rate of recurrence with approximately a six-year total survival of 34–62% [4].

In recent years, modalities of endoscopic pictures have been considered as the standard processes for effective screening of people for proposer diagnosis of laryngeal cancer in the initial phase throughout the entire clinical setting [5]. Such kinds of screening are extensively applicable before performing clinical surgery for histological tissue evaluation in the setting of optical surgery. A few of the approaches such as grouping of CE (contact endoscopy) along with NBI (narrow-band imaging) may demonstrate an improvised and magnified visualization of alterations within the morphology, including 3D alignment of the vocal folds in real time for subepithelial blood vessels [6]. Such kinds of vascular architecture of visualized assessment in CE–NBI pictures may offer complementary datasets for effective clinical treatment of patients suffering from laryngeal cancer across the world [7]. 

**Figure 1:**
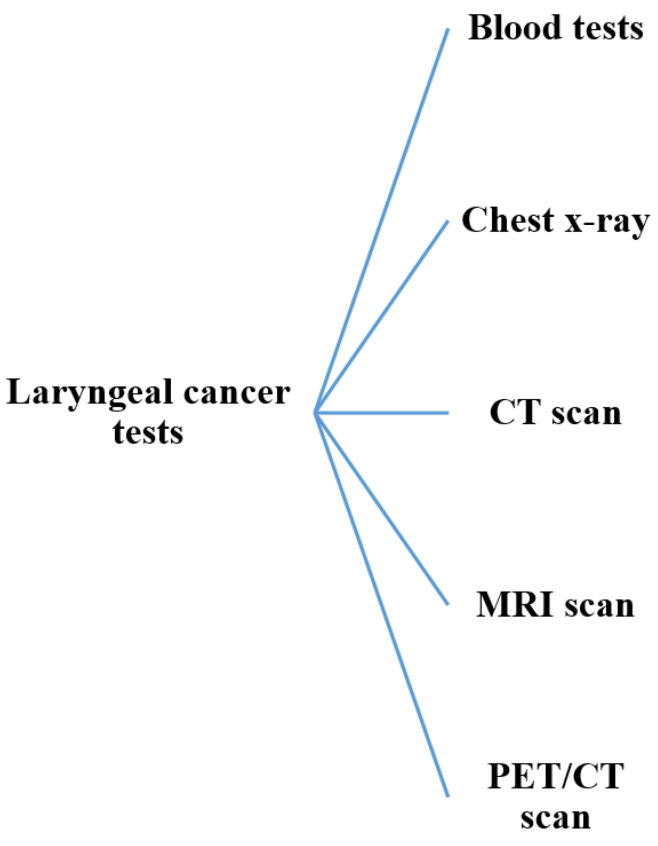
Several of the most common current tests preferred by clinicians for laryngeal cancer identification.

Furthermore, effective utilization of CE–NBI pictures in early prognosis majorly depends upon the otolaryngologist’s practice that demands long years of training in a significant manner [8,9]. Moreover, this may produce outcomes in the crucially subjective choice procedure following under-treatment processes or even over-treatment plans. Currently, advanced-level feature engineering expansion, DL (deep learning), and ML (machine learning) techniques in the field of healthcare application offer multifarious pathways for assisting medical experts and overcoming these kinds of hurdles within clinical trial settings in real time [10,11]. In this context, multiple computer-rooted schemes have been utilized over larynx endoscopic pictures. Such approaches may aid otolaryngologists by offering complementary datasets about the cancer stage along with the vascular tree’s main characteristics and the larynx portion of the entire epithelial tissue [12,13]. Figure 1 illustrates a few of the most common current tests preferred by clinicians for laryngeal cancer identification. 

Laryngeal cancer is a severe disease that is currently and rapidly spreading around the world. Therefore, effective and early diagnosis is becoming essential. According to one report, laryngeal cancer, which is related to head and neck cancers, demonstrates the fifth most common disease across the globe, with approximately 120,000 recent cases and 520,000 deaths in the year 2020. In the present era, laryngeal cancer growth is based on multifarious factors [14]. A few of the most general risk elements involve alcohol intake and smoking, particularly in developed nations around the world. Laryngeal cancer is a huge heterogeneous illness with remarkably diverse diagnoses and therapeutic alternatives that depend on the exact position and extension of the tumor. For the aforementioned intention, preoperative enactment plays a critical role in diagnosis scheduling, which is constantly becoming one of the greatest sufferer-tailored diagnosis methods [15]. 

Currently, PET (positron emission tomography), MRI (magnetic resonance imaging), and contrast-improved CT (computed tomography) pictures are routinely obtained in the entire staging and prognosis procedure of neck and head cancer individuals [16,17]. This immense dataset collected via a plurality of the picture’s modalities within the existing medical data could sizably facilitate investigative radiomic evaluation. This heterogeneous structure of neck and head cancers could be seized through non-invasive procedures, which may assist as a crucial adjunct for medicinal choice making in real time. The major contributions of our work for laryngeal cancer detection are as follows.

- In this work, the authors designed a novel and improvised model for laryngeal cancer detection in the real-time prognosis of patients in an effective manner.
- Another objective of this study is to utilize diverse datasets for testing and cross-validation of the proposed model for laryngeal cancer early identification with a higher accuracy level for quick treatment of patients as per the requirements.
- Our proposed deep-learning-based model was designed by utilizing the enhanced Mask R-CNN technique for improved segmentation of the images for identification of laryngeal cancer with pragmatic accuracy and precision level in real time.
- Furthermore, this improvised model is capable of functioning on ImageNet dataset images along with live CT imaging captured during patient screening, thus accurately identifying patients who have laryngeal cancer or its associated symptoms, or no laryngeal cancer.
- Moreover, this proposed model can be applied to daily healthcare practice and can assist clinicians in effectively reducing the patient screening burden.

## 2. Related Work 

In [17], K. Yamaguchi et al. proposed a novel model for laryngeal cancer detection based on a CNN (convolutional neural network). These days, identification of laryngeal cancer and its associated symptoms is becoming more challenging in the early stages. Laryngeal cancer is generally identified at the progressive phase, patient diagnosis in such a situation is complicated, and possibilities of recovery are low in comparison to identification in the initial stage. In this work, the authors propose a CNN-rooted diagnosis model that is further rooted in a single-shot multi-box detection structure. This model has been trained to utilize the 5164 endoscopic pictures of laryngeal cancer in real time. One of the major limitations of this model is that it is more time-consuming in the case of large picture datasets processed in real time. In [11], J. Unger et al. discussed a new non-invasive scheme for initial-stage discrimination of pre-cancerous vocal fold lesions and malignant laryngeal cancer dynamic assessment in real time. Many laryngeal cancers and their associated symptoms are produced within the vocal cords of people. At present, the early-phase identification of changes in malignant vocal folds of people, along with pre-malignant lesion discrimination, depicts a huge issue within laryngology. Furthermore, tiny carcinomas and lesions of pre-cancerous vocal folds create challenging tasks to differentiate via normal endoscopy procedures in real-time diagnoses. However, this scheme has a few limitations in the modern era, which are related to model accuracy and time consumption in picture pre-processing. 

In [18], N. M. Abd Raboh et al. proposed another scheme for determining an effective amount of tumors budding within the present laryngeal carcinoma in real-time assessment. Tumor budding has become an auspicious diagnostic indicator within numerous types of cancer, such as laryngeal and particularly colorectal cancer. In this work, the authors conducted a retrospective investigation of 118 diverse laryngeal cancer-affected people. The datasets of the patients were acquired via the pathological laboratory records of Ain Shams University’s medical department. However, this study was performed on small datasets and does not provide a pragmatic and detailed evaluation for further investigation in this area. In [19], H. Xiao et al. discussed another study for the recognition of diverse immune genes within laryngeal pre-cancerous cells. The major aim of this study is the recognition of the prognostic immune-type genes and the development of a novel prognostic prototype for the identification of the laryngeal class of cancer that is rooted upon such genes. The authors opted for the medicinal dataset of diverse patients from the Atlas database for the analysis of laryngeal cancer. However, the proposed approach is incapable of processing large datasets for laryngeal cancer identification, and the error rate is higher for larger datasets, which require further investigation for novel methods. 

In [20], S. Kavya et al. discussed another approach for the identification of chances of laryngeal cancer and its related symptoms by utilizing the MFCC (mel-frequency cepstrum coefficients) evaluation. The evaluation was performed through a patient voice coefficient comparison. The accuracy of the model was evaluated based on only 60 samples. One of the major restrictions of this model is the huge pre-processing time of large images for laryngeal cancer detection. In [21], P. Huang et al. discussed another approach to recognize laryngeal cancer in the initial phase. In this work, the researchers chose the CNN method for model development to accurately analyze laryngeal cancer. Further, the authors carried out multiple trials for the testing and validation of the suggested model in real time and compared the performance parameters with the existing approach. In this method, the accuracy was enhanced by 25% when compared to the previous model. However, in the modern technological era, where a large number of datasets are produced on a daily basis for medicinal diagnoses, this suggested model is inefficient for accurate image pre-processing to efficiently organize the datasets. 

In [22], H. Yamazaki et al. presented another article on radiotherapy treatment for an effective cure for laryngeal cancer. In this article, the authors described major technical and certain alternate fractionations. Moreover, the researchers provided an overview of radiotherapy antiquity via 60 cobalt toward recent linear accelerators and placed major emphasis on the function of the alternate fractionation. In [23], the authors proposed a new scheme for the removal of laryngeal cancer utilizing narrow-band endoscopic videos in the real-time screening of patients. In this article, the authors designed and implemented a novel model that determines laryngeal cancer and its associated symptoms from real-time endoscopic videos to save time. However, this procedure is time-consuming, as the videos consume more storage space and take time in information retrieval in the case of large datasets. In [24], F. Wu et al. discussed another method for the picture categorization of laryngeal cancer by using a neural network. In this work, a hybrid model was developed utilizing the ResNet and an inception model for classifying the laryngeal cancer pictures. However, the performance of the model was found to be minimal in the case of fast picture pre-processing and analysis. Table 1 demonstrates the existing work conducted on laryngeal cancer detection techniques. 

## 3. Proposed Methodology 

Effective diagnosis of laryngeal cancer is a critical task, particularly in the initial phase of the development of malignancies in the head and neck region of patients. Earlier diagnosis tools are becoming ineffective in the modern era due to the screening of many people on a daily basis, which is time-consuming for clinicians. For resolving these aforementioned problems, the authors of this work present a novel and more effective approach for patient screening using diverse images. In this work, an improvised approach is proposed for the identification of laryngeal cancer and its related symptoms in real time. This approach is based on a deep learning technique and consumes minimal time in comparison to the previous models. 

### 3.1. Dataset Used

Laryngeal cancer is constantly becoming one of the major threats in people’s lives all around the world. The early detection of laryngeal cancer is important for effective diagnosis and saving the life of the individual, as it is a dangerous kind of cancer among other types of cancers. In this study, the researchers present a new deep-learning-based Mask R-CNN model for the early detection of laryngeal cancer with a greater accuracy level. For the performance analysis of the proposed model, the authors utilized two diverse datasets, namely the ImageNet and multiple CT scan imaging data, in a real-time screening of patients for laryngeal cancer identification [32]. 

#### 3.1.1. ImageNet

ImageNet is one of the most widely utilized picture datasets and is arranged as per the hierarchy of WordNet. Within this hierarchy, all nodes are portrayed via thousands of pictures. This image dataset has been widely utilized by researchers for computer vision advancement and for deep-learning-based research projects. This dataset is accessible to the entire research community for the non-commercial utilization and training and validation of novel models based on multiple new techniques, such as deep learning and many more. 

#### 3.1.2. CT Scan Imaging Datasets

We tested and validated the proposed model using multiple CT scan images captured in the real-time diagnosis of patients by clinicians. The performance computations and results analysis of the proposed model are performed with a greater accuracy level and precision level in real time. CT scan imaging datasets were chosen for cross-validation and testing of the suggested model in a more effective manner. The CT scan used herein is called contrasting CT or may be referred to as contrast-enhanced CT. It is mainly helpful for highlighting structures, namely blood vessels, that are intricate enough for delineating against their surrounding. 

### 3.2. Sample

**Figure 2:**
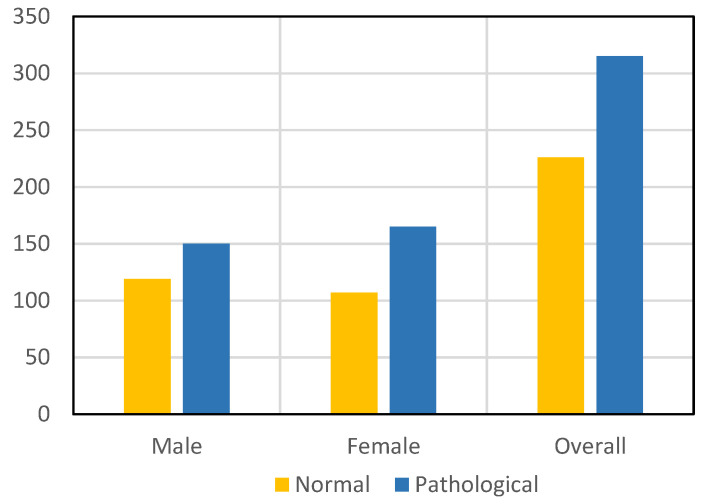
Selected male and female image datasets used for training and validation of the proposed model from the ImageNet database.

For training and validation of our suggested model, we selected both a control group, i.e., health, and a case group, i.e., laryngeal cancer image datasets, for measuring diverse performance parameters such as model accuracy and many more in real time. Further, for accurate training, testing, and model validation, the researchers selected both males and females. For control group images, we selected 119 males and 107 females and an aggregate of 226 image datasets overall in the control group cases. For case group images, we selected 150 male images and 165 case group images and an aggregate of 315 case group pictures overall. Figure 2 illustrates the selected male and female image datasets used for the training and validation of the proposed model.

**Figure 3:**
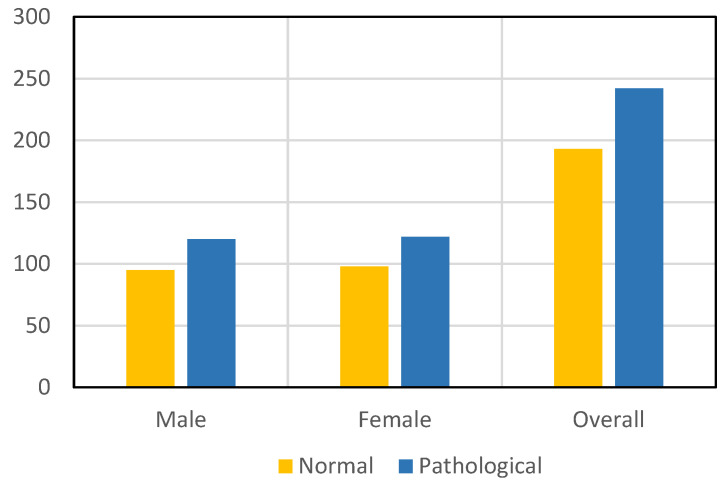
Illustrates the selected male and female images of live CT scan imaging datasets used for training and validation of the proposed model.

Figure 3 illustrates the selected male and female images of the live CT scan imaging datasets used for training and validation of the proposed model. For cross-verification of the proposed model, we selected 95 male and 98 female control group pictures, and an aggregate of all control group pictures was 193 in training and validation. Further, we selected 120 male and 122 female case group images and an aggregate of 242 pictures used for the training and validation of the proposed model in real time. 

### 3.3. System Configuration

The authors performed this experiment with the help of a computer system inscribed with the subsequent system arrangements: 12th generation Intel Core i7 processor, NVIDIA RTX 3050 Ti Graphic card: 4 GB, 16 GB: DDR5 RAM (random access memory), 1 TB SSD (Solid State Drive), Operating System: 64 GB along with Windows 11. For the training of the datasets, we opted for the V3 network of GoogLeNet Inception, which was considered as the backbone network. Furthermore, we utilized StandardScaler (function), which is the globally acknowledged scikit-learn library. This suggested model was designed and validated by utilizing the widely acknowledged framework TensorFlow Keras. 

### 3.4. System Design

**Figure 4:**
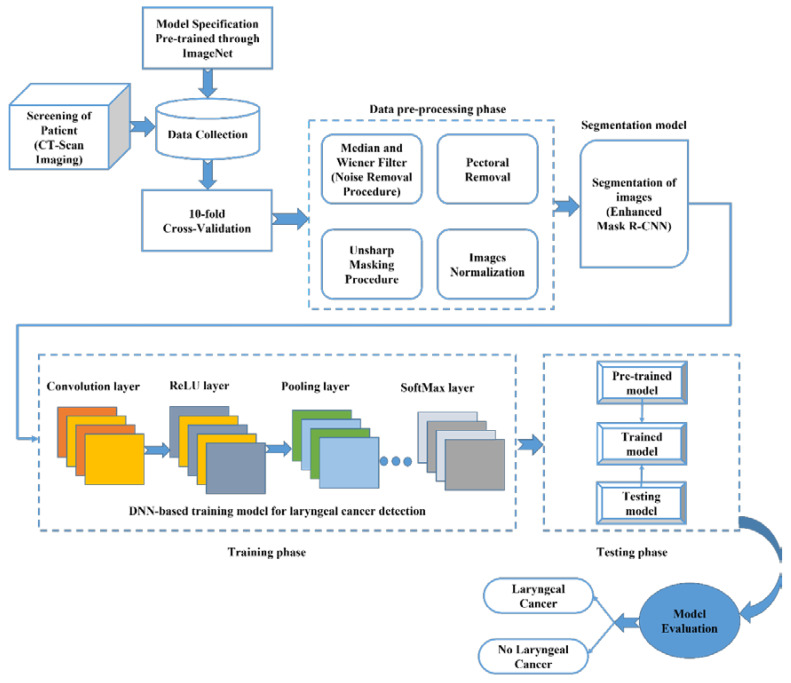
Proposed IML-CNN model for the assessment of laryngeal cancer using image datasets.

In this proposed work, the authors designed a novel model for laryngeal cancer identification using the image datasets for twice the screening of patients in an effective manner. Figure 4 illustrates the proposed IML-CNN model for the assessment of laryngeal cancer using the image datasets. The entire working procedure of this suggested model is described as follows. In this testing procedure, initially, the screening of the patient is performed by using computed tomography (CT) scan imaging for the collection of multiple images of the patient in an effective manner for the diagnosis procedure. At the same time, for testing and verification of the model, the authors utilized the model specification pre-trained through the ImageNet database. We utilized a 10-fold cross-validation approach for the performance computation of the proposed IML-CNN model. The 10-fold cross-validation procedure is a resampling approach that is utilized for the evaluation of the proposed IML-CNN model on a finite dataset sample. The 10-fold cross-validation general procedure is as follows. Initially, the entire collected data were arbitrarily shuffled, followed by division of these data into 10 groups. For every selected distinctive group (a) the group was selected as the testing dataset or hold out; (b) the remaining groups were selected as the training dataset. In the subsequent step, the entire collected dataset was translated for the data pre-processing phase. For effective dataset pre-processing, multiple elements were incorporated by the researchers, including the Median and Wiener Filter for noise removal, from the obtained datasets images. Once the noise was eliminated from the received datasets, after the pectoral removal, unsharp masking and an image normalization process were performed for further image segmentation. 

In the image segmentation procedure, an enhanced Mask R-CNN was utilized. Mask R-CNN is one of the most pragmatic picture segmentation procedures and is based on the CNN technique. The present deep neural network (DNN) variant determines multiple objects within the picture and originates the higher-quality segment masking for every instance in real time. After an effective segmentation procedure, the training phase was performed utilizing the segmented images. This incorporated DNN-based training model contained multifarious hidden layers, which included a convolution layer, ReLU layer, pooling layer, and SoftMax layer for effective categorization of the received pictures. Once the training phase was completed using the DNN-based training model for laryngeal cancer identification, the output was applied to the testing-phase-trained model in real time. Within the testing phase, we integrated three diverse kinds of models, namely the pre-trained, trained and testing models. The pre-trained model was utilized for comparing the acquired datasets in real time, and based upon that, the trained model predicts the precise quality of the applied images. Once the testing procedure is completed, then the model computes laryngeal cancer and no-laryngeal cancer most effectively and accurately. 

The typical architecture of the CNN is generally built through multiple input and output layers that further include diverse essential layers, namely the convolution layer, ReLU layer, pooling layer, SoftMax layer, and finally, the fully connected layers. All of the aforementioned layers are vital for facilitating efficient learning of the higher abstract key features through the applied input datasets, namely the picture in real time. Once the input is applied within the CNN architecture, then the convolution layer obtains the applied input picture for features extraction in an automated manner and sends the obtained datasets to the ReLU layer. The ReLU layer aids in the prevention of exponential progress within the computation needed for neural network operation. These extracted features are to be translated within the pooling layer, and then, the next process is carried out. In the subsequent step, every compact input picture is fed within a fully connected layer for classifying the pictures in the equivalent labels. Finally, the entire obtained dataset is translated into the SoftMax layer to obtain an outcome in a required manner according to the model implementation in real time. The CNN approach has become one of the most recognized and significant kinds of neural networks. The CNN technique overcomes the drawbacks of conventional fully connected layers, which includes but is not limited to computational overhead and more complexity in real-time implementation. 

In our proposed model, for identification of laryngeal cancer and its symptoms in a quick manner with a more accurate outcome, we opted for a hybrid deep-learning-based approach. Here, the input is diverse, and there is no category label for the testing image data, the class label depicts the group of diverse pictures, and lastly, FP represents the list of testing picture datasets. The output here is the list of the trained and validated diverse class picture datasets. The pseudo-code for the proposed enhanced Mask R-CNN segmentation is described below.

**Step 1:** CT scan imaging datasets and ImageNet dataset are aggregated.
**Step 2:** Initialize from CT scan imaging datasets (‘FX’: testing datasets without the label of category) and position this within the combined dataset list.
**Step 3:** Initialize from ImageNet datasets (‘FY’: testing datasets without the label of category) and position this within the combined dataset list.
**Step 4:** Repeat the previous steps until input datasets are within the defined goals (GL1 and GL2).
**Step 5:** Terminate loop and regress ‘failed’ if dataset collection list is detected as unfilled.
**Step 6:** Translate ‘FX’ and ‘FY’ from the chosen dataset list and send to the Median and Wiener Filter (here, entire noise removal and pectoral removal procedures are performed).
**Step 7:** Perform unsharp masking and image normalization of ‘FX’ and ‘FY’.
**Step 8:** Separate and categorize trained datasets with validated diverse classes datasets ‘FX’ and ‘FY’.
**Step 9:** Perform training of categorized datasets ‘FX’ and ‘FY’ in multiple layered DNN-based training model.
**Step 10:** Perform testing of obtained datasets ‘FX’ and ‘FY’ using trained model (here, obtained ‘FX’ and ‘FY’ are trained using a pre-trained model and testing model)
**Step 11:** Predict and evaluate the model outcomes, i.e., laryngeal cancer or no laryngeal cancer.

The source code for the proposed model is also available in the GitHub repository at https://github.com/pravatmca/RCNN/ (accessed on 6 November 2022) for public reference.

### 3.5. Model Development and Features Extraction

The features of the pictures were extracted from the volume of interest (VOI) regions described throughout the segmentation. Each feature demonstrates a particular segment from the entire radiomic notion. The obtained picture intensity was quantified through the histogram intensity, which demonstrates three-dimensional (3D) fractional volume datasets. This received dataset comprises amounts such as overall volume, area of surface, and level of compactness, including the real form of laryngeal cancer in real-time evaluation. The texture-rooted features were compiled through a numerical algorithm, which provides second-order statistical matric characteristics. Diverse features contained in the datasets related to the surplus quality of pictures, such as brightness level, grey shades, and diverse settings, and many more. One of the major encounters in radiosondes is to describe the multiple non-redundant groups of the picture biomarkers through a large number of removed features for improving overall radiometric performance in real time. 

The overall network weights were fixed in both the training and the testing phases using the enhanced Mask R-CNN-based model, and overall loss was evaluated utilizing Equation (1).

(1) $$Overall\;Loss=w_{1}\times rpm\_class\_losses+w_{2}\times rpm\_class\_losses$$

Evaluating the effective segmentation procedure in the initial phase would assist in determining whether there is a need for additional cropping steps of the selected image datasets in real time. There is another performance evaluation metric, namely a disc score, for the medicinal picture segmentation, and it is associated with the Jaccard score by utilizing Equation (2).

(2) $$DS=\frac{2J}{1+J}$$

There is a stringent localization description within the prior part that is utilized as an intersection in comparison with the union, which is also recognized as the Jaccard score. This IOU is given in the Equation (3).

(3) $$\mathrm{IOU}=\frac{\left|A\right|\cap \left|B\right|}{\left|A\right|\cup \left|B\right|}$$

In the case of an estimated boundary box, moved or altered in the dimension, the need for bigger padding would not create a similar IOU. For evaluation of the minimal IoU needed for any given padding dimension, Equations (4) and (5) may be used to calculate ${\mathrm{IOU}}_{Outside}$ and ${\mathrm{IOU}}_{Inside}$.


(4) $${\mathrm{IOU}}_{Outside}=\frac{Dim-X}{Dim+X}$$

(5) $${\mathrm{IOU}}_{Inside}=\frac{Dim^{2}-2Dimx+X}{Dim^{2}}$$

where *Dim* represents the selected dimension of the picture, and *X* represents the padding dimension. 

### 3.6. Performance Metrics

The performance evaluation of our proposed deep-learning-based model was conducted by utilizing accuracy, recall, precision, and F1 score as the main performance metrics. Each of the aforementioned performance metrics along with the corresponding mathematical formula is described as follows.

The accuracy metric is simply the ratio of accurately categorized image samples to the overall amount of the samples as depicted in Equation (6).

(6) $$Accuracy=\frac{TP+TN}{TP+TN+FP+FN}.$$

The accurate value of precision is a measurement of the ratio of accurately evaluated diverse images to the overall amount of positive categorization forecasts as depicted in Equation (7).


(7) $$Precision=\frac{TP}{TP+FN}$$

The recall metrics are simply a ratio of the real positive, accurately predicted image classes as described in Equation (8).

(8) $$Recall=\frac{TP}{TP+FN}$$

The F1 score metrics are an average of both the recall and precision as depicted in Equation (9).

(9) $$F1\mathrm{score}=\frac{2\times Recall\times Precision}{Recall+Precision}$$

## 4. Results and Discussion

Laryngeal cancer is becoming one of the biggest threats to people’s lives all around the world. This category of cancer characterizes one of the greatest recurrent cancers within the neck and head regions of people. Laryngeal cancer identification in the early stage is still a challenging problem for clinicians, as traditional detection methods are time-consuming and are incapable of identifying laryngeal cancer and its symptoms in the early phases. The larynx demonstrates distinctive diagnostic and therapeutic issues for medical experts during patient screening. Laryngeal cancer’s intricate area and diverse structure make this essential for improving clinical screening diagnosis instruments to enhance the choice-making protocols for higher survival rates and functional outcomes. Therefore, there is a massive need for a novel and cost-effective model that can be implemented more easily and conveniently in comparison to the previous scheme in real time. In this work, the researchers proposed a novel and enhanced model for the identification of laryngeal cancer and its associated symptoms in the early stage in a short amount of time and in a more accurate manner in comparison to the existing models. For these reasons, this suggested model is a pragmatic choice to monitor and evaluate laryngeal cancer and its symptoms, particularly in surgical and non-surgical tissue-safeguarding treatments. 

In our work, a fully automated deep-learning-based model was proposed for more accurate laryngeal cancer identification and classification. In this improvised model, we utilized the enhanced Mask R-CNN approach for a more practical categorization of malignant and benign laryngeal cancer. To the best of our knowledge, there has been no work performed on the identification of early-stage laryngeal cancer using the enhanced Mask R-CNN approach, which provides more accurate classification results of laryngeal cancer and its associated symptoms in the early stage, which could save the lives of more patients through accurate diagnosis. The outcome of the proposed model demonstrates that the suggested deep-learning-based model is capable of distinguishing benign lesions from diverse shapes and sizes of malignant lesions from both the ImageNet datasets and the live CT scan imaging datasets. Furthermore, the suggested model offers greater performance constraints and more consistent clarification along with an objective choice-making procedure for medical experts during the screening and diagnosis of patients. The application of our proposed deep-learning-based model has brought more efficient results in the field of picture evaluation for a better understanding of diverse medical picture contents. Furthermore, the proposed model is capable of reutilizing the pre-trained model, namely for picture categorization in real-time analyses. This experiment was performed by utilizing the widely recognized framework TensorFlow Keras on a computing machine with the following system arrangements: 12th generation Intel Core i7 processor, NVIDIA RTX 3050 Ti Graphic card: 4 GB, 16 GB: DDR5 RAM, 1 TB SSD, Operating System: 64 GB and Windows 11. For the training of both datasets, the researchers utilized the V3 network of GoogLeNet Inception, which was considered as the backbone network. 

**Figure 5:**
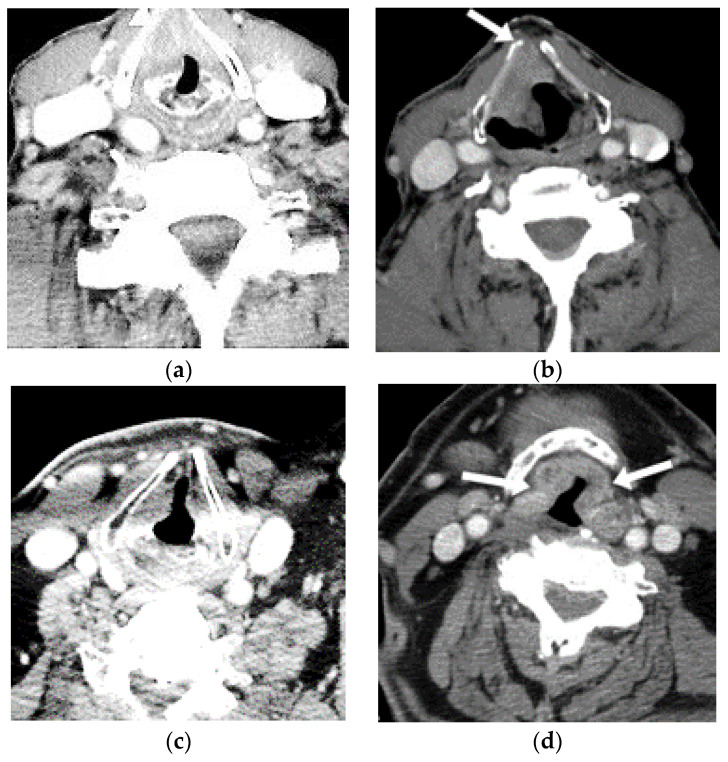
Illustrates the selected training and testing pictures. (**a**) Original training picture, (**b**) tested image with clearer white spots depicting laryngeal cancer, (**c**) original training picture, and (**d**) another tested picture with clearer white spots depicting laryngeal cancer.

Figure 5 illustrates the selected training and testing pictures: (a) shows the original training picture, (b) shows the tested image with clearer white spots depicting laryngeal cancer, (c) demonstrates the original training picture, and (d) shows another tested picture with more clear white spots depicting laryngeal cancer. This attention map is portrayed as a heat mapping overlain on real pictures. The warmer colors indicate greater saliency, which shows a huge contribution to the categorization choices. The outcome of the proposed model is pragmatic and offers clearer imaging for the identification of laryngeal cancer in comparison with the existing approaches, and it can help clinicians in the early diagnosis of patients. Thus, this work offers a more accurate and visible screening of patients in real time and does not leave the minimal or maximal condition of laryngeal cancer and its associated symptoms in the medical screening procedure. 

**Figure 6:**
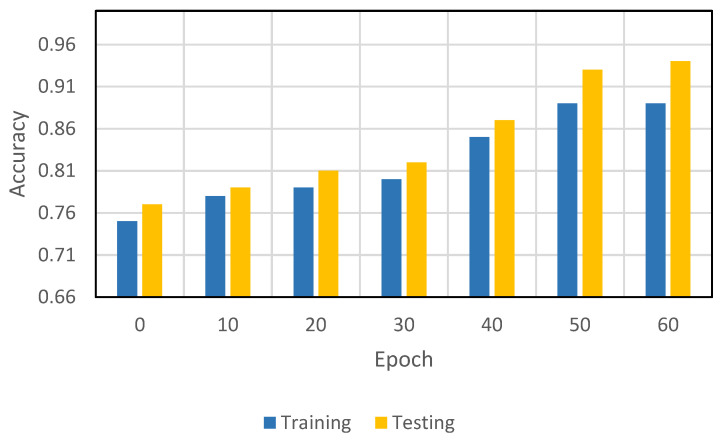
Accuracy of the proposed model both in the training and testing phases, taking into account live CT scan imaging datasets.

Figure 6 illustrates the accuracy of the proposed model both in the training and testing phases, taking into account live CT scan imaging datasets. The measured training and testing accuracy of the proposed model on epochs 0, 10, 20, 30, 40, 50, and 60 are 0.75, 0.78, 0.79, 0.8, 0.85, 0.89, 0.89 and 0.77, 0.79, 0.81, 0.82, 0.87, 0.93, 0.94, respectively. Our proposed model accuracy on the CT scan imaging datasets is improved and more practical in comparison to the existing models. 

**Figure 7:**
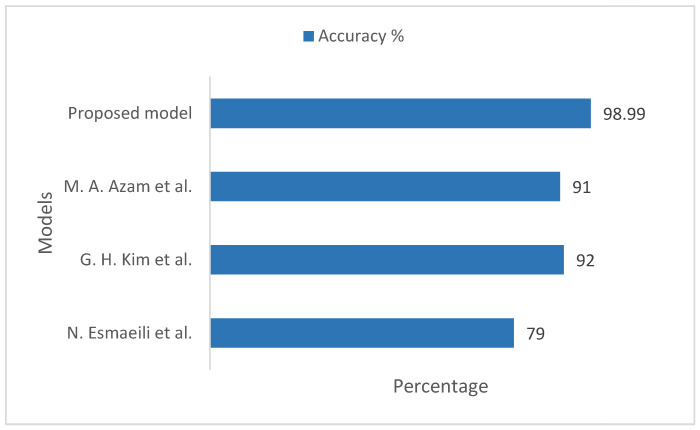
Comparison of accuracy between the proposed model and existing models, taking into account the ImageNet datasets [33,34,35].

Figure 7 illustrates the comparison of accuracy between the proposed model and the existing models, taking into account the ImageNet datasets. For the existing models by N. Esmaeili et al. [33], G. H. Kim et al. [34], and M. A. Azam et al. [35], the measurement accuracy was 79%, 92%, and 91%, respectively. Our proposed model measured training and validation accuracy on the ImageNet datasets to be 98.99%, which is more practical and pragmatic in comparison to the existing approaches. Furthermore, our model works efficiently on large image datasets regarding diverse gender in a more accurate manner and assists clinicians in effective screenings of patients in real time. 

**Figure 8:**
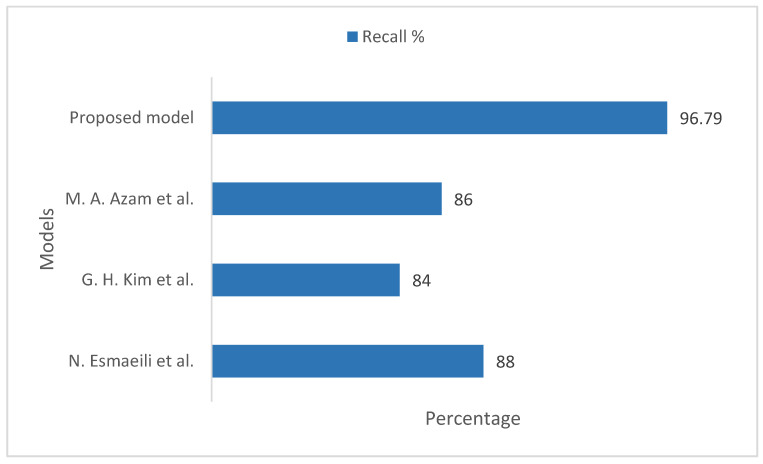
Recall comparison of the proposed model with existing models, taking into account the ImageNet datasets [33,34,35].

Figure 8 illustrates the recall comparison of the proposed model with existing models, taking into account the ImageNet datasets. In the existing models, N. Esmaeili et al. [33], G. H. Kim et al. [34], and M. A. Azam et al. [35] showed measured recall values of 88%, 84%, and 86%, respectively. Our proposed model measured the training and validation recall values on the ImageNet datasets to be 96.79%, which is more realistic and considerable in comparison to the existing models.

**Figure 9:**
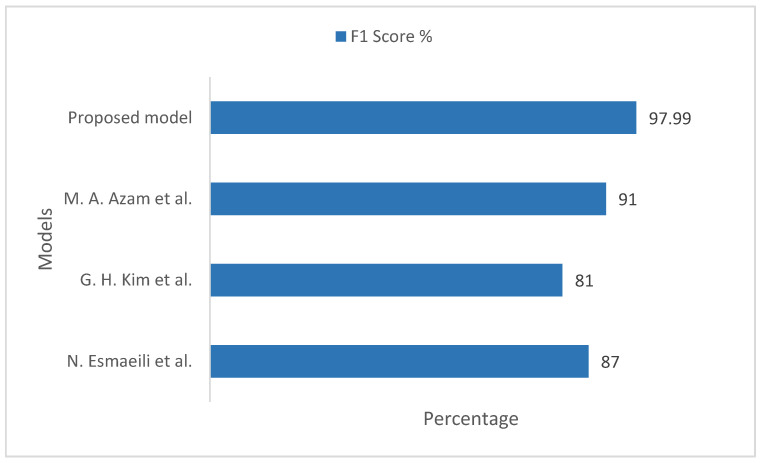
Comparison of F1 score between the proposed model and the existing models, taking into account the ImageNet datasets [33,34,35].

Figure 9 illustrates the F1 score comparison of the proposed model with the existing models, taking into account the ImageNet datasets. In the existing models, the measured F1 score values were 87%, 81%, and 91%, respectively. Our proposed model measured training and validation F1 score value on the ImageNet datasets to be 97.99%, which is more realistic and considerable in comparison to the existing models.

**Figure 10:**
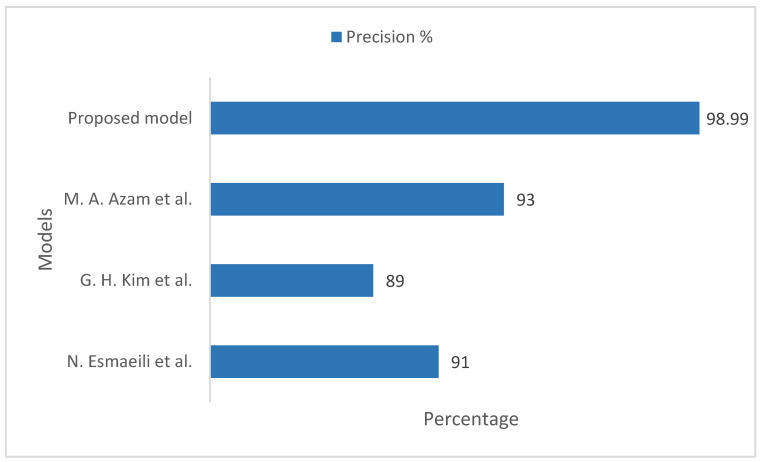
Comparison of precision between the proposed model and existing models, taking into account the ImageNet datasets [33,34,35].

Figure 10 illustrates the precision comparison of the proposed model with existing models, taking into account the ImageNet datasets. In the existing models, the measured precision values were 91%, 89%, and 93%, respectively. Our proposed model measured the training and validation precision value on the ImageNet datasets to be 98.99%, which is more realistic and considerable in comparison to the existing models.

**Figure 11:**
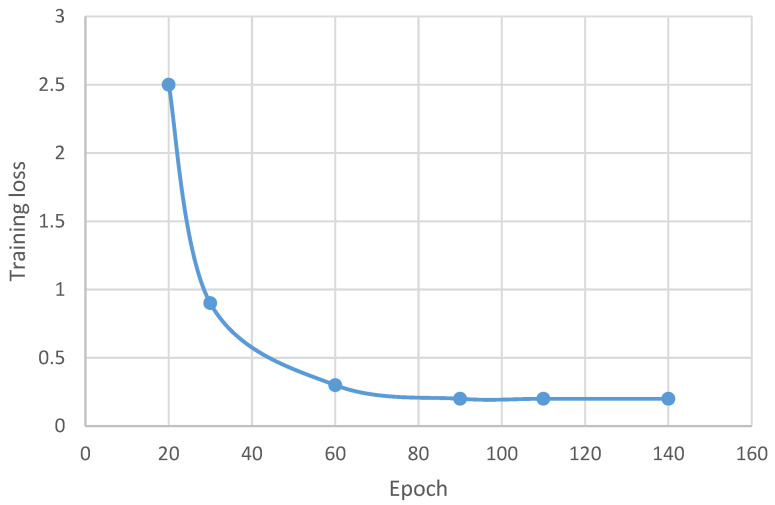
Training loss of the proposed model.

Figure 11 illustrates the training loss of the proposed model. The training loss of the proposed model measured on the diverse chosen epochs 0, 20, 30, 60, 90, 110 and 140 are 0%, 2.5%, 0.9%, 0.3%, 0.2%, 0.2% and 0.2%, respectively. The outcome of the proposed model using the deep learning approach offers minimal training losses in comparison to the previous approaches. 

**Figure 12:**
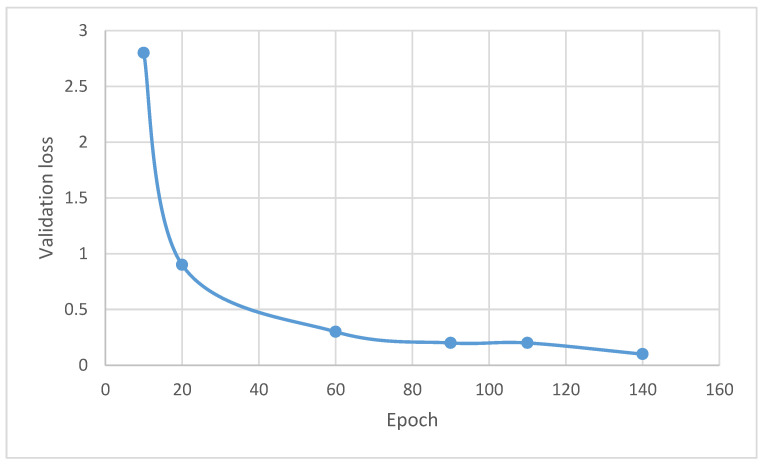
Validation loss of the proposed model.

Figure 12 illustrates the validation loss of the proposed model. The validation loss of the proposed model measured on the diverse chosen epochs 0, 20, 30, 60, 90, 110 and 140 are 0%, 2.8%, 0.9%, 0.3%, 0.2%, 0.2% and 0.1%, respectively. The results of the suggested model utilizing the deep learning approach offer minimal validation losses in comparison to the previous approaches. 

Table 2 depicts the comparative analysis of the time of execution between the proposed model and the existing models. The time of execution of any model is a critical parameter and requires much attention toward minimization of time consumption during the training and validation of the proposed model in real-time analysis of the model. The execution times for the existing models for laryngeal cancer identification were 7, 5, and 4 s, respectively. Our proposed model time of execution both in the training and validation phases was 1 s, which is minimal in comparison to the previous models. Thus, our model’s minimal execution time demonstrates that it is capable of handling and processing a huge amount of datasets for laryngeal cancer identification in a more pragmatic manner. 

## 5. Conclusions

The laryngeal cancer identification and categorization procedure is critical work for clinicians, especially in the initial development phases of benign and malignant lesions within the head and neck portion of patients [36]. The larynx of the individual is one of the greatest multifaceted anatomical and essential organs inside the head and neck area. In the last decade, cases of laryngeal cancer among people have been constantly increasing across the globe, owing to diverse reasons such as more alcohol consumption, smoking and an unhealthy diet in day-to-day life. An effective and accurate diagnosis in the initial development phase is essential for saving the lives of people, as laryngeal cancer is a severe disease. In this paper, the authors developed a novel and improvised deep-learning-based model for laryngeal cancer detection and identification, especially in the initial phase. Our proposed model is one of the novel and most suitable models in comparison to previous models because it is capable of identifying laryngeal cancer and its associated symptoms with more accuracy and in a minimal time duration using the diverse images datasets. In summary, we developed an automated and well-suited model for clinical analysis and for more accurate screening of patients for the identification of laryngeal cancer. This model was developed in such a manner by utilizing diverse components so that it could use a variety of image datasets such as the ImageNet and live CT scan imaging datasets for laryngeal cancer identification. The model also shows considerable medicinal impact, along with enhanced diagnosis rates in the development of early laryngeal cancer. The results of the suggested deep-learning-based model are improved and offer an accuracy of 98.99%, precision of 98.99%, F1 score of 97.99%, and recall of 96.79% on the ImageNet dataset. Moreover, the proposed model took only 1 s for clinical screening both in the training and validation of the diverse image datasets, which is minimal and could help endoscopists and other medical experts in the medical testing of patients in real time. In the future, there is great potential and possibilities for more investigations to detect laryngeal cancer by using diverse datasets for minimizing the time of screenings and improving the accuracy of the model, especially on larger datasets.

## Figures and Tables

**Table 1:** Existing work conducted on laryngeal cancer detection techniques.

S. No.	Name of Author	Reference	Publication Year	Technique Used	Accuracy Level (%)	Limitation
1	M. Bengs et al.	[25]	2020	CNN	81%	Large computation time in classification
2	G. B. Gour et al.	[26]	2020	SVM and RF	76%	Higher computation complexity in implementation
3	E. Hadjaidji et al.	[14]	2021	SVM along with KNN	83%	Lower classification accuracy on large datasets
4	X. Zhou et al.	[27]	2021	VGG16, and ResNet50 classifier	85%	Model prediction speed is lower in real time
5	T. M. Inbamalar et al.	[28]	2021	CNN	86%	Incapable of processing and training large images datasets in the modern era
6	M. H. Laves et al.	[29]	2019	DCNN	84%	Low accuracy level
7	M. Żurek et al.	[30]	2022	AI-based multimodal prediction model	91%	Large power consumption and lower throughput
8	Q. Cen et al.	[31]	2019	CNN	88%	More computational complexity in multiple datasets

CNN: convolutional neural network, AI: artificial intelligence, DCNN: deep convolutional neural network, KNN: K-nearest neighbors, RF: random forest, SVM: support vector machine, ResNet: residual network.

**Table 2:** Comparative analysis of the proposed model and existing model time of execution.

S. No.	Models	Time of Execution (in Seconds)
1	N. Esmaeili et al. [33]	7 s
2	G. H. Kim et al. [34]	5 s
3	M. A. Azam et al. [35]	4 s
4	Proposed Model	1 s

## Data Availability

The dataset used in this paper is publicly available [32].
